# Hemorrhagic risk prediction in coronary artery disease patients based on photoplethysmography and machine learning

**DOI:** 10.1038/s41598-022-22719-7

**Published:** 2022-11-10

**Authors:** Zhengling He, Huajun Zhang, Xianxiang Chen, Junshan Shi, Lu Bai, Zhen Fang, Rong Wang

**Affiliations:** 1 State Key Laboratory of Transducer Technology, Aerospace Information Research Institute, Chinese Academy of Sciences, Beijing, China; 2 University of Chinese Academy of Sciences, Beijing, China; 3 Department of Cardiovascular Surgery, The Sixth Medical Centre of PLA General Hospital, Beijing, China; 4 Beijing UniStrong Science & Technology Co., Ltd, Topscientific Systems Inc, Beijing, China

**Keywords:** Data mining, Machine learning, Diagnosis

## Abstract

Hemorrhagic events are the main focus of attention during antithrombosis therapy in patients with coronary artery disease (CAD). This study aims to investigate the potential of using photoplethysmography (PPG) and machine learning techniques to assess hemorrhagic risk in patients with CAD. A total of 1638 patients with CAD were enrolled from January 2018 to October 2019, among which 114 patients were observed to have at least one positive event. Importantly, 102 patients with 9933 records were finally retained for analysis in this study. Participants were required to collect data using the portable PPG acquisition device and the specially designed Android APP. The data was collected and uploaded to a remote server. Based on collected PPG signals, we extracted features in a total of 30 dimensions from time-domain, frequency-domain, and wavelet packet decomposition. Logistic regression, support vector regression, random forest, and XGBoost regression models were established to achieve hemorrhagic risk evaluation, and then, their performances were compared. In total, 10 features extracted from PPG showed statistical significance (*p* < 0.01) between negative and positive groups. The newly established XGBoost model performed best in the hemorrhagic risk evaluation experiment, wherein the mean area under the curve (AUC) with tenfold cross-validation was 0.762 ± 0.024 and the sensitivity and specificity were 0.679 ± 0.051 and 0.714 ± 0.014, respectively. We established a data acquisition system for PPG signal collection, and demonstrated that a set of features extracted from PPG and the proposed machine learning model are promising in the evaluation of hemorrhagic risk among patients with CAD. In comparison with the traditional HAS-BLED score, the proposed method can obtain the quantitative risk prediction probability from a single PPG record, which has the advantages of dynamics and continuity, and can provide timely feedback for doctors' antithrombotic treatment, which is of great significance for doctors to quickly determine the effectiveness of the treatment and adjust the timely treatment plans accordingly.

## Introduction

Coronary artery disease (CAD) is considered the leading cause of death in humans, and according to the World Health Organization, it has the highest fatality rate in the world. Implementing antithrombotic therapy, such as anticoagulant and antiplatelet agents^1,2^, is important for patients with CAD, as it can significantly reduce the incidence of early and long-term adverse cardiovascular events. However, various hemorrhagic events related to antithrombotic therapy are also increasing day by day, which has become an important concern during antithrombotic therapy^3,4^. This is because the occurrence of these events leads to the termination of treatment and then leads to new hemorrhagic events, thus resulting in disability and even death. Therefore, predicting bleeding risk during antithrombotic therapy and identifying sensitive factors related to the outcomes of these events are quite valuable.

The HAS-BLED score is an important and popular method for assessing the bleeding risk during anticoagulant therapy for patients with CAD. It is widely used because of its simplicity and reliability^5^. The score includes seven factors such as hypertension, abnormal renal and liver function, stroke, bleeding history, labile international normalized ratio, elderly (such as age over 65 years), and consumption of drugs or alcohol. Patients with a low risk of bleeding are classified as those with a score of 0–2, whereas patients with an increased risk of bleeding represent a score of ≥ 3. However, a patient's bleeding risk is often constantly changing, so the HAS-BLED score lacks dynamics and continuity. In addition, the HAS-BLED score relies on clinical variables and has only seven risk factors, so it lacks more perspectives to characterize the physical condition. Therefore, how to carry out long-term and dynamic monitoring of patients' physical states and make more accurate predictions of hemorrhagic events are more realistic but more challenging.

Photoplethysmography (PPG) is utilized to reflect the blood movement from the heart to the peripheral tissues (e.g., a finger in the present study) and is composed of alternating current (AC) and direct current (DC) components. The AC component is attributed to changes in blood volume generated by each heartbeat, whereas the DC component is mainly regulated by the activity of the breathing, sympathetic nervous system, and thermoregulation^6^. PPG is a low-cost and noninvasive optical technique for cardiovascular system evaluation. It carries information related to the state of blood vessels and hemodynamics^7^ and can quantitatively assess changes in the state of the cardiovascular system using morphological characteristics of the PPG waveform^8^, such as oxygen saturation^9^, noninvasive cuff-less blood pressure estimation^10,11^, screening tests for deep vein thrombosis^12^, large artery stiffness^13^, the study of venous hemodynamics of the lower limb^14^, and detection of hypovolemia^15^. With the advent of integrated circuits and microelectronics, PPG measurements can be integrated with more portable, compact wearable devices to provide low-cost and continuous monitoring in the home environment^16^. To the best of our knowledge, there are no studies using PPG for predicting the risk of bleeding, especially during antithrombotic therapy.

The purpose of this study was to explore the potential of using PPG and machine learning techniques to assess the bleeding risk in patients with CAD. We have established an online data acquisition system that enables users to collect PPG signals at home and upload the data to a central database. First, we preprocessed the collected data and evaluated the signal quality. Then, we extracted the 30 dimensional features from the time domain, frequency domain, and wavelet packet decomposition (WPD), and we finally established machine learning models to predict the bleeding risk. This method has characteristics of continuity, dynamic, and precision and is a useful addition to the existing HAS-BLED score method, providing a new approach and means for the prediction of hemorrhagic risk during antithrombotic therapy.

## Methods

### Study design

This study has been approved by the medical ethics committee of Chinese People's Liberation Army General Hospital (Approved number: s2017-044-02), and the Chinese Clinical Trial Registry number is ChiCTR1900028125. All study methods were carried out in accordance with relevant guidelines^17,18^, and all participants provided written informed consent. A total of 1638 patients with CAD were enrolled from January 2018 to October 2019. The inclusion and exclusion criteria were as follows:

- Inclusion criteria:
  1. Patients with the age of 18 ≤ 80, with no gender limitation;
  2. Patients diagnosed with CAD, including patients who underwent percutaneous coronary intervention and coronary artery bypass grafting, and patients receiving medical therapy;
  3. Patients who have clearly understood the study through consultation, voluntarily participated in the study, and signed the consent and equipment loan agreement.
- Exclusion criteria:
  1. Patients with systemic active infection;
  2. Patients with severe hepatic and renal insufficiency (ALT > 135 mmol/L and Cr > 200 µmol/L);
  3. Patients with chronic obstructive pulmonary disease;
  4. Patients with obvious bleeding tendency and blood diseases;
  5. Patients with malignant tumors and end-stage diseases;
  6. Patients with heart valve or left ventricular aneurysm and other simultaneous surgery;
  7. Patients with cerebrovascular diseases who have had cerebral infarction or cerebral hemorrhage currently or within the last 6 months;
  8. Patients who have recently participated in an investigational drug study and have not completed the primary end point or have interfered with the clinical end point.

Devices for PPG acquisition were distributed to each participant for free after they were instructed on how to properly use them. All participants were required to take a measurement once a day. In particular, prior to starting the measurement, each patient rested for 5 min in a quiet environment, and then, the measurement process sustained for 60 s. Upon completion of the measurement, the user was asked to fill a form on APP to record if there was an end point hemorrhagic (or bleeding) event. Finally, the original PPG signal and the filled form were uploaded to the remote server via HTTP protocol for further processing, and the PPG signal, record time, event label, and patient ID were synchronously stored in the database. There was a total of 10 different clinical end point events, including subcutaneous hemorrhage, gingival bleeding, epistaxis, hematochezia, black stool, hematemesis, hematuria, retinal hemorrhage, cerebral hemorrhage and other hemorrhagic events. We categorized all of these events into the positive group. If no positive event occurred, it was categorized into a negative group, and then, we considered this task as a binary classification problem.

### Data acquisition

The data acquisition device was implemented by Beijing UniStrong Science & Technology Co., Ltd., Topscientific Systems Inc. The working voltage of the device is 3.5 V, and it works in transmission mode generated infrared light from an LED with a wavelength of 885–895 nm. The signal is digitized by the detector with a sampling rate of 500 Hz. The depicted PPG signal is transmitted to the Android APP in real-time via Bluetooth 4.0 link.

### Data preprocessing

**Figure 1:**
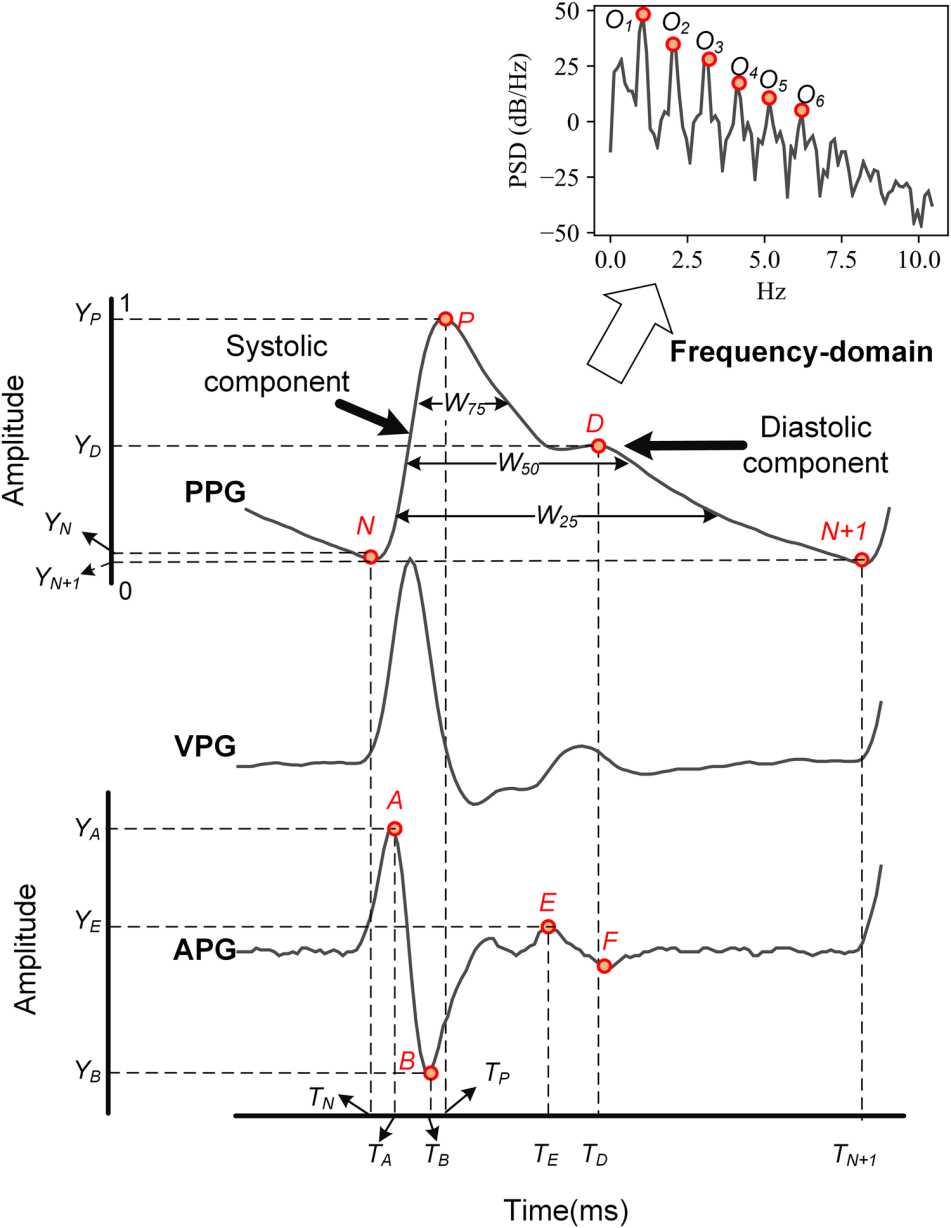
PPG, the first derivative of PPG (VPG), the second derivative of PPG (APG) signal and their features. The abbreviations *T* and *Y* denote the time (ms) and amplitude of the corresponding points, respectively.

One heartbeat PPG waveform consisted of systolic and diastolic components, including the systolic notch (*N*), systolic peak (*P*), and diastolic peak (*D*), as shown in Fig. 1. The record length from each patient was 60 s with three different luminous intensities, and we only used the 20 s corresponding to the medium luminous intensity. A Butterworth band-pass filter with a cut-off frequency of 0.2 Hz and 20 Hz was then applied to remove the baseline wandering and high-frequency noise, respectively. An algorithm proposed by Zong et al.^19^ was used to detect the systolic notch (*N*) of each PPG waveform.

### Features extraction

Njoum et al. have performed a simulation study and demonstrated that PPG also had the potential to be used as a simple noninvasive method for the detection of blood characteristics^20^. In addition, the second derivative of the PPG (APG) is also widely used in hemodynamic-related research. APG represents the acceleration of blood flow under the measurement position, and it contains valuable information according to reported literature^21,22^. Its main advantage is that it can eliminate the influence of baseline shift^23^ and provide stable information for the irregular shape of PPG^24^. Therefore, analysis of the PPG signal was not limited to the original PPG signal in this study, and the first derivative of the PPG (VPG) and APG were also included. The synchronized PPG, VPG, and APG signals are shown in Fig. 1. A 30 dimensional feature vector was extracted based on these three types of waveforms from the time-domain, frequency-domain, and wavelet packet decomposition, as shown in Table 1.

**Table 1:** Definition of extracted features from PPG and APG.

Type (count)	Feature name	Definition
Time-domain (14)	Tp, Td, HR	*T*_*P*_*-T*_*N*_*, T*_*D*_*-T*_*N*_*, 60,000/(T*_*N*+*1*_*-T*_*N*_*)*
	RI, SI, T_RD	*Y*_*D*_*/Y*_*P*_*, Height/(T*_*D*_*-T*_*P*_*), (T*_*P*_*-T*_*N*_*)/(T*_*N*+*1*_*-T*_*P*_*)*
	Rslop, Dslop	*(Y*_*P*_*-Y*_*N*_*)/(T*_*P*_*-T*_*N*_*), (Y*_*P*_*-Y*_*N*_*)/(T*_*N*+*1*_*-T*_*P*_*)*
	Rarea	$\int_{{T_{N} }}^{{T_{P} }} {Y_{PPG} dt} /\int_{{T_{P} }}^{{T_{N + 1} }} {Y_{PPG} dt}$
	VDab, VDae	*Y*_*A*_*/Y*_*B*_*, Y*_*A*_*/Y*_*E*_
	W25, W50, W75	*Pulse width at the 25%, 50% and 75% height of the systolic peak, respectively*
Frequency-domain (5)	H1–H5	*Normalized harmonic power in the frequency domain, using Welch's method (O*_*2*_*/O*_*1*_–*O*_*6*_*/O*_*1*_*)*
Wavelet packet decomposition energy features (11)	E1–E10, Eall	*E1*–*E10: Normalized energy obtained by wavelet packet decomposition, from 0 to 9.77 Hz (0.977 Hz for each); Eall: The total energy of PPG signal (0*–*250 Hz)*

#### Time-domain features

Point *N* of each PPG waveform was first detected by the algorithm mentioned above. Considering that points *A, B,* and *P* are all local extreme points, they can be obtained by applying appropriate searching windows on point N. Point *D* corresponds to the closure of the aortic valve or the end of blood ejection^25^, generally, it can be determined by positive to negative zero-crossings point on VPG. However, a large number of PPG have inconspicuous diastolic notches and diastolic peaks. In this case, we first located the local maximum point *E* on the APG, which represents the maximum local acceleration of blood flow, indicating the arrival of the diastolic component. When acceleration becomes the local minimum (point *F*), the PPG approximately reaches the diastolic peak, so we used mapping of point *F* on PPG as an alternative of point *D*. Finally, a total of 14 time-domain features can be extracted, their definitions are shown in Table 1, the abbreviations T and Y denote the time (ms) and amplitude of the corresponding points, respectively. It should be noted that the amplitude of each waveform has been first normalized to the range of 0–1 before extraction of features.

#### Frequency-domain features

The PPG signal consists of rich components in frequency. In comparison with time-domain analysis, frequency-domain analysis is more robust and can be less affected by interferences. We used Welch's method to obtain the power spectral density of the PPG signal^26^. Specifically, The PPG signal $X = \{ x_{0} ,x_{1} ,...,x_{N - 1} \}$ was divided to $K$ segments with length $M$, and the overlap size was set to $S$, so the $k{ - }th \, \left( {k = 1, \ldots ,K} \right)$ segment was $X_{k} = \{ x_{(k - 1)S} ,...,x_{(k - 1)S + M - 1} \}$. Defined a window function $W = \{ W_{0} ,...,W_{M - 1} \}$ for the current segment. For frequency component $v = i/M$ with $1 - M/2 < i < M/2$, a windowed discrete Fourier transform (DFT) can be calculated:

1 $$X_{k} (v) = \sum\limits_{{m{ = }0}}^{M - 1} {X_{k,m} W_{m} \exp ( - 2j\pi vm)}$$

Forming the periodogram value for each segment:

2 $$P_{k} (v) = \frac{1}{{\sum\nolimits_{{m{ = }0}}^{M - 1} {W_{m}^{2} } }}|X_{k} (v)|^{2}$$

Welch’s estimate of the PSD can be obtained by averaging, and we converted the unit to decibel (dB):

3 $$PSD(v) = 10*\lg \left(\frac{1}{K}\sum\limits_{k = 1}^{K} {P_{k} (v)}\right )$$

In this study, $M$ was set to 4096, $S$ was set to 1024 and the “Hanning” window was chosen as the window function $W$. Based on the obtained PSD, we can determine the position of each harmonic via detecting the extreme point, and then the amplitude of the first six harmonics was obtained, in which each harmonic from the second to the sixth was normalized by dividing them by the power of the fundamental frequency (first harmonic). They were considered as the frequency-domain features H1–H5.

#### Energy features based on wavelet packet decomposition

Wavelet decomposition is another time–frequency analysis method that has been widely used in different fields after the fast Fourier transform method^27–29^. Wavelet decomposition first decomposes the signal into high and low-frequency components and then iteratively decomposes the low-frequency signals but maintains the high-frequency components, whereas the WPD also decomposes the high-frequency components of the signal. The decomposition result can be represented as a tree, and sub-band energies are defined as followings:

4 $$E_{j}^{k} = \left| {C_{j}^{k} } \right|^{2}$$

where $C_{j}^{k}$ is the coefficient of frequency band *j* in decomposition level *k*. The total energy is the sum of energy in sub-band:

5 $$Eall = \sum\limits_{j = 1}^{n} {E_{j}^{k} }$$

In this study, WPD was implemented until 8 levels ($n = 2^{8} = 256$), and “sym6” belonging to symlets family was chosen as the mother wavelet^30^. The normalized energy is:

6 $$Ej = \frac{{E_{j}^{k} }}{Eall}$$

In 1983, Lee et al. analyzed the spectral density of the PPG signal, and the ratios between 1–10 Hz and 1–50 Hz were calculated. The results showed that over 99% of the energy of the PPG signal was concentrated in the range of 1–10 Hz^31^. So, we only focused on the frequency components within 10 Hz. After using 8 levels of decomposition, the bandwidth of each sub-band was 0.977 Hz due to the sampling rate being 500 Hz. Nonetheless, 10 components were ultimately reserved as E1–E10, and the total energy of the PPG signal was denoted as Eall.

### Model and evaluation

Logistic regression (LR), support vector regression (SVR), random forest (RF), and XGBoost regression models were established for the classification task. The output probability of the model can be regarded as a hemorrhagic risk score, and the performance of these four models can be compared. These models have been extensively discussed in the existing literature^32,33^. However, a detailed description of their principles is not our goal in this study.

A receiver operating characteristic curve (ROC) was used for model performance evaluation. The sensitivity and specificity are defined as follows.

7 $$Sensitivity = \frac{TP}{{TP + FN}}$$

8 $$Specificity = \frac{TN}{{TN + FP}}$$

where TP, TN, FP, and FN denote true positive, true negative, false positive, and false negative, respectively. ROC can be then depicted by the above two parameters. AUC is defined as the area under the ROC. Generally, an AUC of > 0.5 indicates that the model is more effective than a random one.

Several hyperparameters for the above models need to be adjusted to obtain optimal performance in this task^34^. We used AUC as the target of optimization, and used the grid search method^35^ with five-fold cross-validation to optimize the hyperparameters for each model. The hyperparameters to be optimized and their search range, step and the final optimal value are shown in Table 2.

**Table 2:** Hyperparameters optimization process using the grid search method.

Hyperparameters	Search range	Step	Optimal value
max_depth	3–10	1	7
learning_rate	0.01–0.21	0.05	0.06
gamma	0–0.05	0.01	0.01
subsample	0.5–1.0	0.1	0.7

After determining the parameters, the models were evaluated by tenfold cross-validation, i.e., 90% of the data was used to train the classification model, whereas the remaining 10% of the data was used for testing, and features were normalized using the Z-Score method. In addition, during training, to keep class balance, we used random down-sampling to extract the same number of samples from the negative group as that of the positive group.

### Statistical analysis

Mann–Whitney U test, which has no restrictions on the normal distribution of data, was used to compare two independent groups of samples in this study, wherein data were presented as mean ± SD. A value of *p* < 0.01 was considered statistically significant.

## Results

In the present study, a total of 114 subjects were observed with at least one bleeding event and were defined as positive subjects. The demographic characteristics are given in Table 3.

**Table 3:** Demographic characteristic of patients.

Demographic characteristic	Mean ± SD
Age	61.6 ± 11.2
Height (cm)	167.4 ± 8.2
Weight (kg)	69.9 ± 12.4
SBP (mmHg)	127.4 ± 19.6
DBP (mmHg)	80.3 ± 12.0
Body Mass Index	24.8 ± 3.3

**Figure 2:**
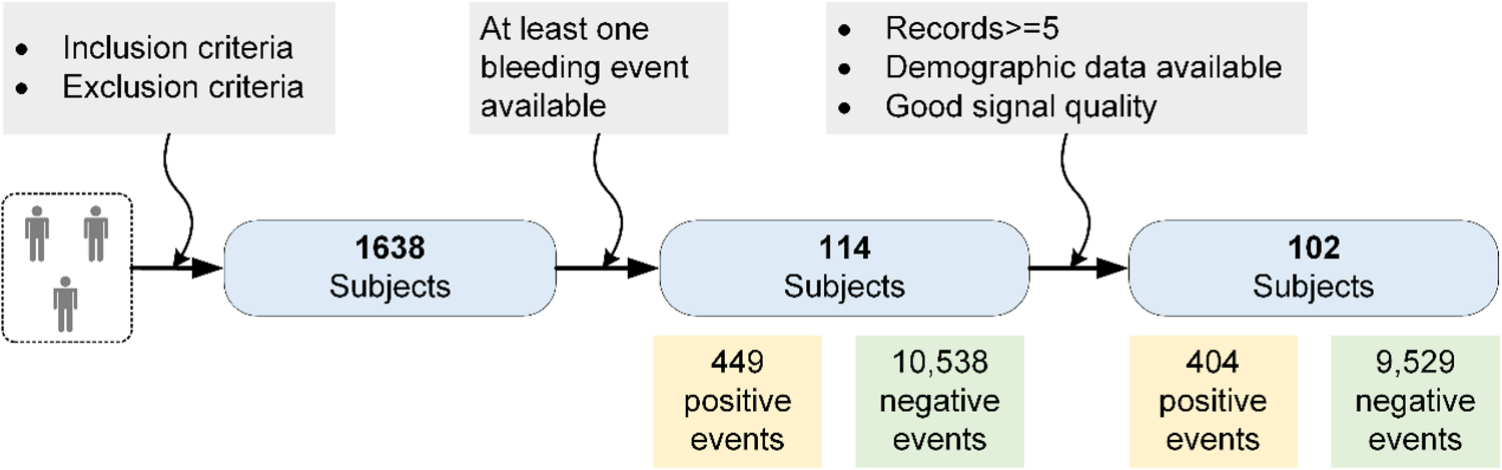
Statistics of subject and record numbers at different stages.

To avoid demographic differences affecting the results, this study included only PPG records of positive subjects during bleeding and nonbleeding periods and did not include all PPG records uploaded by negative subjects. Positive subjects uploaded a total of 449 PPG signals during bleeding periods that were defined as positive events, whereas the other 10,538 PPG signals collected during nonbleeding periods were defined as negative events. First, patients with fewer than five records were directly excluded, and this threshold was empirically determined in this study. These situations generally occurred due to the fact that these users performed the measurements only a few times after the registration but were unable to persist in them in their daily life. Second, records with no demographic data and records with poor signal quality were excluded. Finally, valid data were retained for 102 positive subjects, including 404 positive events and 9529 negative events. Statistics of subject and event numbers at each stage is shown in Fig. 2.

**Figure 3:**
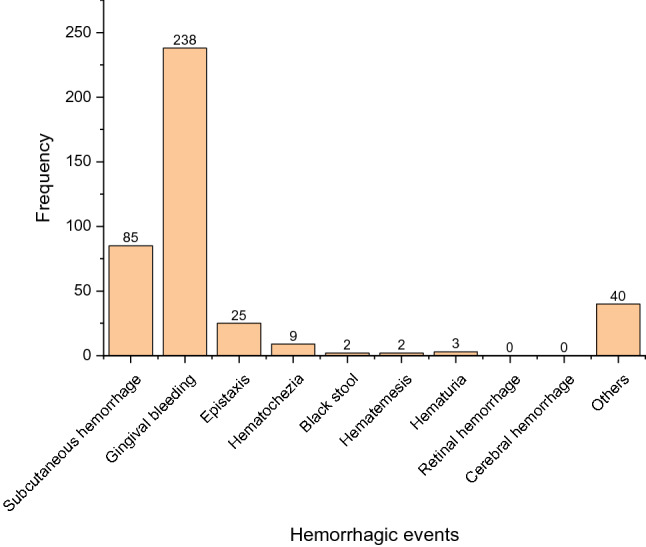
Frequency of hemorrhagic events in positive individuals.

The frequency of positive hemorrhagic events is shown in Fig. 3. It can be seen that the most reported event was gingival bleeding, whereas retinal hemorrhage and cerebral hemorrhage events were not observed during this period.

A comparison of 10 features with significant statistical differences between negative and positive groups is shown in Table 4. It can be seen that Td in the positive group was smaller than that in the negative group, whereas the RI was larger. From the perspective of geometric characteristics of the PPG waveform, it is possible that the diastolic component of the waveform in the positive group is delayed, causing an increase in the width of the diastolic wave and a decrease in Td and Rarea. H1–H5 in frequency-domain were observed to be statistically different between the negative and the positive groups, and the normalized powers of five harmonics were gradually decreasing on a single one. The total energy Eall given by WPD was smaller in the positive group, whereas no significant differences were observed in the other sub-bands. In previous studies, SI, VDab, and VDae were reported as valuable parameters for identifying arterial vascular stiffness^36^. However, no significant difference was found between the SI obtained in the negative and positive groups, i.e., 6.44 ± 1.31 and 6.44 ± 1.16 (m/s, *p* = 0.079), respectively. Besides, VDab and VDae showed no significant differences.

**Table 4:** Comparison of 10 features with significant statistical differences (*p* < 0.01) between negative and positive groups.

Feature name	Negative group (label = 0)	Positive group (label = 1)
Td (ms)	472 ± 45	465 ± 36
RI	0.442 ± 0.103	0.474 ± 0.103
Rarea	0.454 ± 0.106	0.441 ± 0.124
W50 (ms)	328 ± 71	338 ± 72
H1	0.852 ± 0.062	0.838 ± 0.042
H2	0.742 ± 0.078	0.721 ± 0.066
H3	0.580 ± 0.132	0.559 ± 0.088
H4	0.510 ± 0.127	0.482 ± 0.086
H5	0.450 ± 0.130	0.417 ± 0.108
Eall (dB)	92.4 ± 5.9	88.9 ± 7.1

**Figure 4:**
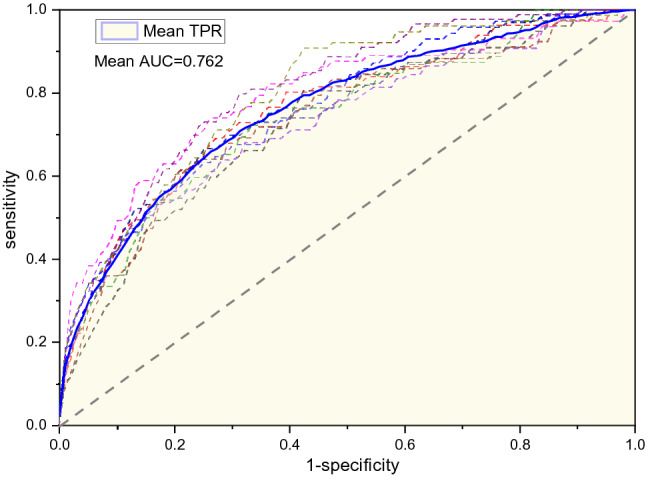
ROCs, mean ROC, and AUC with tenfold cross-validation of the XGBoost model.

The sensitivity and specificity of the four models are shown in Table 5. The mean AUC obtained using the XGBoost model was 0.762. Its sensitivity and specificity were 0.679 ± 0.051 and 0.714 ± 0.014, respectively, which were significantly higher than other models and random prediction results as well, showing the effectiveness of the proposed features and model. Its ROCs, mean ROC, and AUC with tenfold cross-validation are shown in Fig. 4.

**Table 5:** Sensitivity and specificity of the four models.

Model	Sensitivity	Specificity
LR	0.646 ± 0.056	0.683 ± 0.015
SVR	0.634 ± 0.053	0.703 ± 0.028
RF	0.646 ± 0.061	0.709 ± 0.024
XGBoost	**0.679 ± 0.051**	**0.714 ± 0.014**

Significant values are in bold.

## Discussion

PPG has shown its value in predicting cuff-less blood pressure, deep vein thrombosis, etc. in cardiovascular system-related research. Therefore, investigating the potential of PPG to assess the hemorrhagic risk in patients with CAD can be of interest. In the present study, we have established a data acquisition system that can easily collect PPG signals from multiple users in a distributed manner. Based on the analysis of PPG signals collected in a large-scale manner in the past 2 years, 30 features were finally extracted and four machine learning models were established and validated. To the best of our knowledge, this is the first time that PPG has been applied to hemorrhagic risk evaluation tasks. Nonetheless, its high compliance, noninvasiveness, and portability make it a promising tool.

### Physiological basis of the proposed features

According to previous research, structural and functional changes in blood vessel walls and vascular stiffness increase the risk of bleeding^37,38^. Specifically, arterial stiffness accelerates the transmission of pulse waves and also allows more energy to be transmitted from the heart to the periphery. Excessive force may result in rupture of small arteries at multiple sites, which is manifested as breakage and atrophy of the elastic lamina, fragmentation and dissections of vascular smooth muscle, or granular or vesicular cellular degeneration. Acampa et al. have found that arterial stiffness is a possible pathogenetic factor that modifies arterial wall properties, and it contributes to vascular rupture in response to an acute elevation in intravascular pressure during in-depth intracerebral hemorrhage^37^. Ding et al. found that carotid arterial stiffness may contribute to the pathophysiology of cerebral microbleeds, especially at deep locations^38^.

The morphology (wave shape), intensity (wave amplitude), speed (wave speed), and rhythm (wave periodicity) of the PPG waveform can be used to comprehensively reflect the current state of the human cardiovascular system. RI and SI are the most widely used indicators for PPG contour analysis, wherein they quantify the reflected waves in terms of amplitude and time, respectively. A diastolic peak is due to the reflection of the pressure wave by arteries of the lower body back to the finger^39^, increased vascular stiffness causes increased resistance that leading to a decrease in the compliance of the elastic arteries, and causing an earlier return of the diastolic peak. Consequently, this process increases left ventricular after load and impairs coronary perfusion^36,40^. In the present study, RI is smaller and Td is larger in the negative group, which means that the diastolic wave arrives later in the negative group, indicating better vascular compliance. However, we have not observed significant differences in SI, VDab, or VDae. In other similar studies, Millasseau et al. analyzed the effects of different doses of nitroglycerin (GTN) on RI and SI, where the results showed that the effects of GTN on RI were more significant than that on SI. One of the reasons for this is that vasoactive drugs have more significant effects on the vascular tone of small muscular arteries than on the stiffness of the large arteries, which ultimately leads to the differences between amplitude and reflection time^13^, resulting in different behaviors of RI and SI. In addition, Takazawa et al. also studied the effects of drugs and age on APG parameters, and it was found that VDab did not change significantly after the use of angiotensin or nitroglycerin, but did change with age^41^.

Harmonic analysis is another widely used PPG analysis approach. Huang et al. proposed a harmonic energy ratio to analyze the arterial pulse spectrum and found that the energy in the fourth to the sixth harmonic of patients with palpitation was significantly less than that in the normal subjects. They used blood circulation (redistribution of blood flowing into the organs) to explain the results and illustrated the relevance to Traditional Chinese Medicine (TCM) theory, which demonstrated the major importance of harmonic analysis^42^. Wang et al. also established a connection between TCM theory and harmonic analysis and discussed the relation to "pulse feel"^43^. Hsiu et al. found that harmonics of blood pressure and PPG were significantly changed in a cold stimulation (CS) experiment, indicating that the responses of harmonic indexes can be used to quantify the sympathetic reactivity to CS and the resultant arterial stiffness^44^. In addition, Sherebrin et al. demonstrated that the power of harmonics decreased with age or arteriosclerosis^45^. Kern et al. used PPG harmonics for human blood pressure estimation^46^. In their opinion, the phase velocities of the fundamental and higher harmonics of PPG depend on the (nonlinear) elastic properties of the artery. Therefore, the phase velocity varies with the instantaneous dilation of blood vessels and can be used effectively for blood pressure estimation. In the present study, we observed that both the negative group and the positive group showed a trend of gradual decline from H1 to H5, and in terms of harmonics of the same order, the negative group was higher than the positive group. We inferred that due to the existence of risk factors that induce bleeding, the positive group suffered greater energy loss during the blood conduction process, which was reflected in the reduction of the PPG harmonic amplitude.

The above harmonic analysis can reveal the frequency and energy characteristics of the PPG waveform. To further study the energy of each sub-band, we introduced WPD to decompose PPG signals into different frequency bands, thereby capturing the information at fine granularity. Energy features such as E1-E10 and Eall were proposed, and larger energy values were observed in the negative group, as shown in Table 4. It is worth noting that Eall showed a significant statistical difference in the comparison between the two groups. This trend was in good agreement with the results we observed from the harmonic features, that is, the energy features observed in the positive group were generally smaller than those observed in the negative group, indicating that the blood in the positive group suffered greater energy loss during the blood conduction process, and thus, the PPG energy obtained from the finger was smaller.

### Classification model performance

XGBoost is an extreme gradient boosting machine that boosts numerous weak predictors into a strong one with higher precision. Weak predictors are based on a tree model, and they are required to give more precision than random guessing^47^. XGBoost has quickly become one of the most popular machine learning algorithms in various fields of health care, such as atrial fibrillation detection^48^, and evaluation of physical fitness level^49^, owing to its higher performance and speed in comparison with other ensemble learning algorithms. In the present study, XGBoost finally achieved an average AUC of 0.762, which was higher than LR, SVM, and RF models, proving the feasibility of XGBoost in hemorrhagic risk assessment and further expanding its application to the health care field. XGBoost effectively mined information related to the outcome event from PPG features, and it obtained an accurate prediction probability through a PPG record. In comparison with the HAS-BLED model, this model has the advantages of continuity and dynamics and can provide a new and efficient means for hemorrhagic risk prediction.

### Importance of features

**Figure 5:**
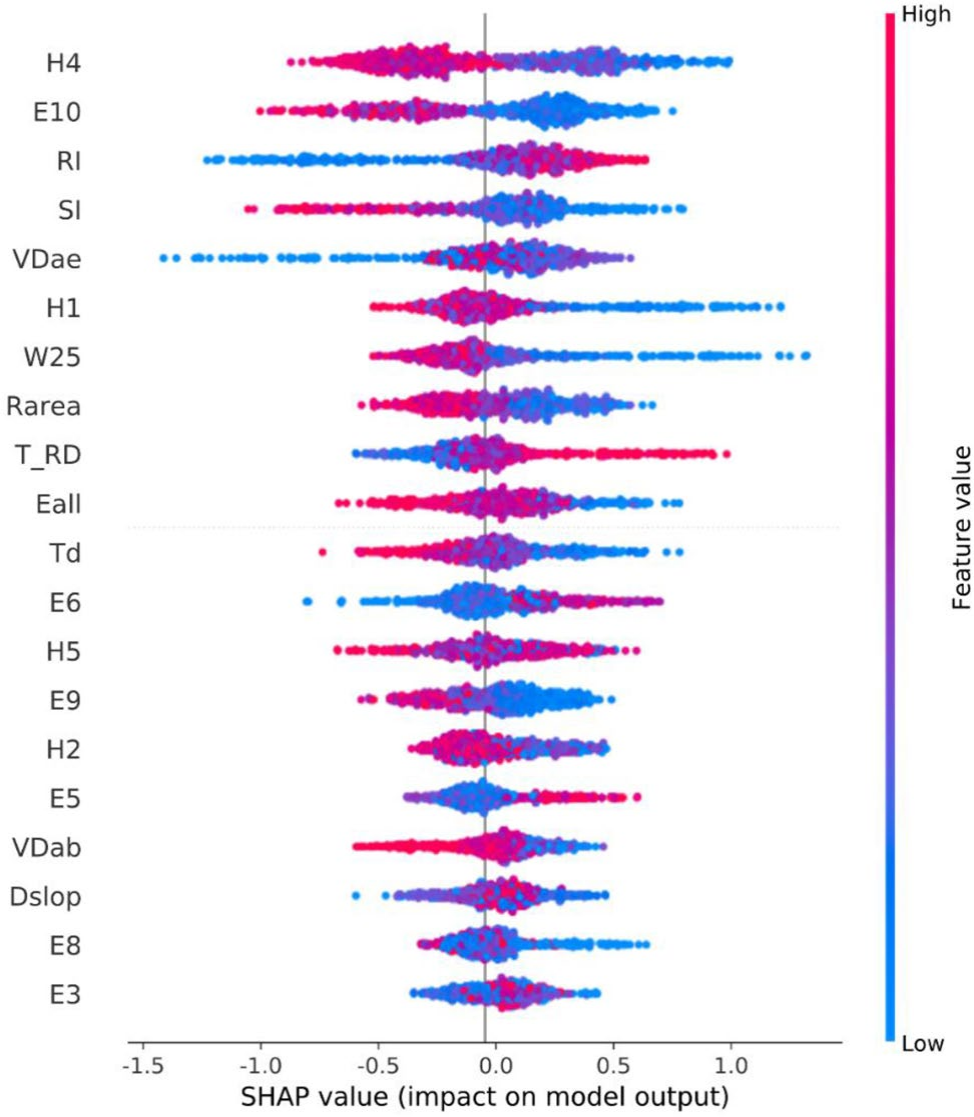
Feature importance analysis by SHAP framework.

We further used the SHapley Additive exPlanations framework (SHAP)^50,51^ to analyze the importance of features of the XGBoost model. However, only 20 features were displayed, as shown in Fig. 5. The SHAP value represents the contribution of the feature to the target (negative: 0 and positive: 1). The feature value is represented by different colors, i.e., the larger the feature value, the redder the color. On the contrary, the smaller the feature value, the bluer the color. For example, with the decrease in H4, E10, SI, and so on, the SHAP value tends to be positive, which means that the model is more inclined to identify the current sample as target 1, indicating that the smaller these features, the higher the risk of bleeding. On the contrary, with the increase in RI, VDae, and so on, the SHAP value tends to be positive, indicating that the greater these features, the higher the risk of bleeding. According to such a principle, the results presented in Fig. 5 tend to be consistent with the results in Table 4. For example, for the negative group with low bleeding risk, Td, Rarea, H1, H2, H4, H5, and Eall have higher values, whereas for the positive group with high bleeding risk, RI is relatively higher, indicating that the prediction results of the XGBoost model are consistent with traditional statistical analysis methods. In addition, the sum of the absolute value of the SHAP value reflects the importance of the feature, so H4, E10, RI, SI, and some other features can be considered as the relatively important ones according to this criterion. However, it should be emphasized that this study was carried out on a relatively small dataset, so a larger dataset is needed to verify the stability of feature importance. In addition, we should avoid excessive emphasis on the importance of a single feature. Since bleeding events can be represented by a variety of features of PPG, it is usually hard to use a single feature to make accurate decisions about results. This is also the main reason why we introduced machine learning algorithms, which can comprehensively consider multiple factors and avoid a single factor to determine the final result, so as to achieve better accuracy and robustness.

### Study limitations

There are limitations to our study that should be considered. At first, patients were asked to perform measurements in a home environment, so the data acquisition mainly depended on user compliance. The total upload time was 131.1 ± 139.3 days among patients, which was actually lower than our expectation. In addition, an insufficient follow-up period may make it difficult to observe possible positive hemorrhagic events in patients. As a result, the overall number of positive events is small, which presents great challenges to the establishment of machine learning models and is also the main reason for the difficulty in further improving the mean AUC. In future work, we plan to prolong the follow-up period for massive data collection to further validate our model and apply deep learning approaches to automatically extract abstract representations from the data without the intervention of human experts^52^, which may further improve the performance and reliability of the risk evaluation model.

## Conclusion

In this study, we established a data acquisition system for PPG signal collection. Based on the acquired data, we extracted 30 features from the PPG signal and established machine learning models for hemorrhagic risk evaluation. A best mean AUC of 0.762 was obtained with optimal parameter selection (grid search method) using the XGBoost model, demonstrating the potential of PPG and machine learning algorithms in hemorrhagic risk evaluation. In addition, we found that 10 features extracted from PPG showed statistical significance between negative and positive groups, and they had the potential to be sensitive hemorrhagic risk factors. This model can be easily deployed on the server, integrated with the remote data collection system, and used to alert patients with CAD to possible hemorrhagic events in their home environment or to assist doctors in selecting therapeutic schemes. In our future work, we will further prolong the follow-up period for massive data collection and explore the application of deep learning to this task to promote this field.

## Data Availability

The dataset included in this study is available from the corresponding author on reasonable request.
